# Inference of Functional Relations in Predicted Protein Networks with a Machine Learning Approach

**DOI:** 10.1371/journal.pone.0009969

**Published:** 2010-04-01

**Authors:** Beatriz García-Jiménez, David Juan, Iakes Ezkurdia, Eduardo Andrés-León, Alfonso Valencia

**Affiliations:** 1 Computer Science Department, Universidad Carlos III de Madrid, Madrid, Spain; 2 Structural Biology and Biocomputing Programme, Spanish National Cancer Research Centre - (CNIO), Madrid, Spain; Michigan State University, United States of America

## Abstract

**Background:**

Molecular biology is currently facing the challenging task of functionally characterizing the proteome. The large number of possible protein-protein interactions and complexes, the variety of environmental conditions and cellular states in which these interactions can be reorganized, and the multiple ways in which a protein can influence the function of others, requires the development of experimental and computational approaches to analyze and predict functional associations between proteins as part of their activity in the interactome.

**Methodology/Principal Findings:**

We have studied the possibility of constructing a classifier in order to combine the output of the several protein interaction prediction methods. The AODE (Averaged One-Dependence Estimators) machine learning algorithm is a suitable choice in this case and it provides better results than the individual prediction methods, and it has better performances than other tested alternative methods in this experimental set up. To illustrate the potential use of this new AODE-based Predictor of Protein InterActions (APPIA), when analyzing high-throughput experimental data, we show how it helps to filter the results of published High-Throughput proteomic studies, ranking in a significant way functionally related pairs. Availability: All the predictions of the individual methods and of the combined APPIA predictor, together with the used datasets of functional associations are available at http://ecid.bioinfo.cnio.es/.

**Conclusions:**

We propose a strategy that integrates the main current computational techniques used to predict functional associations into a unified classifier system, specifically focusing on the evaluation of poorly characterized protein pairs. We selected the AODE classifier as the appropriate tool to perform this task. AODE is particularly useful to extract valuable information from large unbalanced and heterogeneous data sets. The combination of the information provided by five prediction interaction prediction methods with some simple sequence features in APPIA is useful in establishing reliability values and helpful to prioritize functional interactions that can be further experimentally characterized.

## Introduction

A number of computational methods have been developed to predict functional associations in complete genomes. In particular, three such methods have frequently been tested [1] and implemented in well-organized popular servers [2]: phylogenetic profiles (PP, [3]) examine the presence or absence of genes in related species; genomic context (GC, [4]) considers the conservation of the gene neighborhoods (proximity in the chromosome organization) in different species; and gene fusion (GF, [5]–[6]) studies pairs of proteins for which a homolog of each of them has been fused in the same protein. These methods have in common the use of evolutionary information. Moreover, variants of these methods are appearing continuously [7]–[9]. The performance of some variants of these three methods was shown to be similar to some of the High Throughput experimental proteomics approaches when compared to a manually curated gold standard set [1].

Other interaction prediction methods, such as *in silico* two-hybrid (I2H, [10]) and mirror tree (MT, [11]), use multiple sequence alignments and principles of co-evolution. Applying these methods to large data collections produces a considerable number of false positives, which is probably related to the additional evolutionary trends that partially dilute the signal directly related to protein-protein interactions [12]–[14]. However, there is currently significant research activity in this area addressing these problems, with new methodological approaches displaying a better capacity to specifically distinguish the true co-evolutionary information [12]–[14].

Previous studies have addressed the integration of data coming from several sources (mostly experimental) for improving the prediction of protein interactions in *S. cerevisiae* [15]–[16]. These methods rely on the large amount of experimental data available for this organism and are mainly devoted to the assignment of reliability values to experimentally derived protein interactions. In contrast, the work presented here explores the combination of several aspects of evolutionary information (instead of experimental information), in order to discover new protein functional associations (not restricted to protein physical interactions) between protein pairs. Therefore, our approach differs from previous studies both in the *a priori* input information and in its eventual applicability.

In the study presented here, we propose the application of a machine learning methodology to improve the prediction of functional interactions. Our approach is based on the combination of the results of various prediction methods independently developed in this area. We propose the use of a recently published machine-learning algorithm known as “Averaged One-Dependence Estimators” [17] to optimally combine the various prediction methods. Our results show that the performance of the AODE-based predictor is superior to a number of alternative classifiers based on different machine learning algorithms when their results are compared with a carefully derived data collection of *E. coli* functional associations.

Finally, we have used our predictor to refine collections of functional protein associations, including those obtained by a high-throughput experimental approach [18] and interaction predictions extracted from the widely used STRING server [2].

## Results

### Performance comparison for several classifiers

We explored several algorithms and various training sets with different positive/negative ratios in order to reach a good compromise between the actual underlying class unbalance (most protein pairs are not expected to be functionally related). This helped us determine whether the methods tested were suitable to address the incapacity of most algorithms to handle highly unbalanced sets (see the details in Methods).

This exploratory process generated several classifiers, the performance of which had to be compared using our test set of functional protein relationships (see Methods). The comparative analysis was performed using Cost Curves (Curve Tool [19]) that allow us to rapidly choose the best classifier by direct visual inspection. As a rule of thumb, the best classifiers lie below the worst ones because they have a lower cost (classification mistakes). Indeed, the error difference between a pair of classifiers can be measured through the vertical distance between their curves (a brief description of the use of these curves is provided in the Methods section).

**Figure 1:**
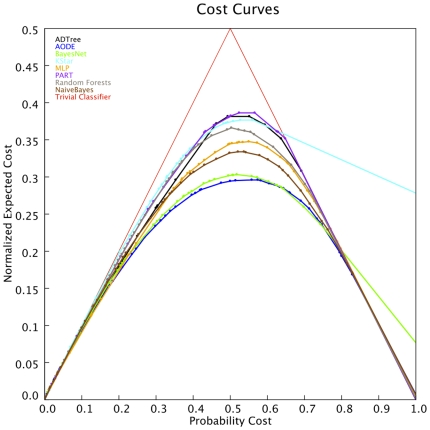
Cost Curves for several machine learning algorithms. The X-axis represents the probability cost and the Y-axis the normalized expected cost. Each cost curve corresponds to a different machine-learning algorithm. Looking at the legend from top to bottom, the algorithms are: ADTree (Alternating Decision Tree); AODE and BayesNet, two Bayesian methods; Kstar, a case based reasoning algorithm; MLP, MultiLayer Perceptron, a neural network; PART, a rules decision method; Random Forests, a combination of classification trees; and Naïve Bayes. The last one is the trivial classifier, without any algorithm assigned. See “Methods/Learning Algorithm” sub-section for the reference of each algorithm.

The Cost Curves for different classifiers representing the different machine learning algorithms used were examined and from a visual inspection of this plot, it was clear that the best performing algorithm in this preparatory phase test was AODE (blue line, Fig. 1). The AODE Cost Curve lies below the probability cost curves of all the other methods at most values, which means that AODE makes fewer mistakes than any other classifier for most positive/negative ratios (false positive and false negative predictions). BayesNet [20]–[21] is the second best classifier in terms of performance, emphasizing that Bayesian-based classifiers are the most appropriate to address this problem. Interestingly, BayesNet scored worse than AODE and even than the trivial classifier when the probability cost was greater than 0.8. This fact will be irrelevant in most of the cases (with very low positives/negatives ratios), although it would become relevant for those experiments implying the filtering of highly reliable sets of associations obtained from experimental sources. Moreover, BayesNet replaces any missing value with the median value from the training set for the corresponding attribute, instead of ignoring this value as AODE does. In this respect the AODE approach is more appropriate for the semantics in our domain where a missing value implies non-existence. This is an important issue because most of the entries have at least one missing value and the information contained in the lack of a value is expected to be more instable and more difficult to extrapolate when predicting on new entries. It is also interesting that the third best classifier according to Cost Curves (Figure 1) is Naïve Bayes (NB) [22] (that considers independence among the input features), the comparison of these three methods show that modeling the internal dependence between the features clearly improves the results. It is important to note that although other algorithms (*eg.* Random Forests [23]) have shown their value on previous related works [15], AODE is shown to be more appropriate for the explored combinations of problem, features and experimental system. Therefore the superior performance of AODE for this preparatory phase test cannot be taken as a proof of the general superiority of the algorithm. Different methods are expected to yield different results according the specific characteristics of each prediction problem. Moreover, some of these classifiers could improve their behavior by exploring in detail their parameter space.

**Figure 2:**
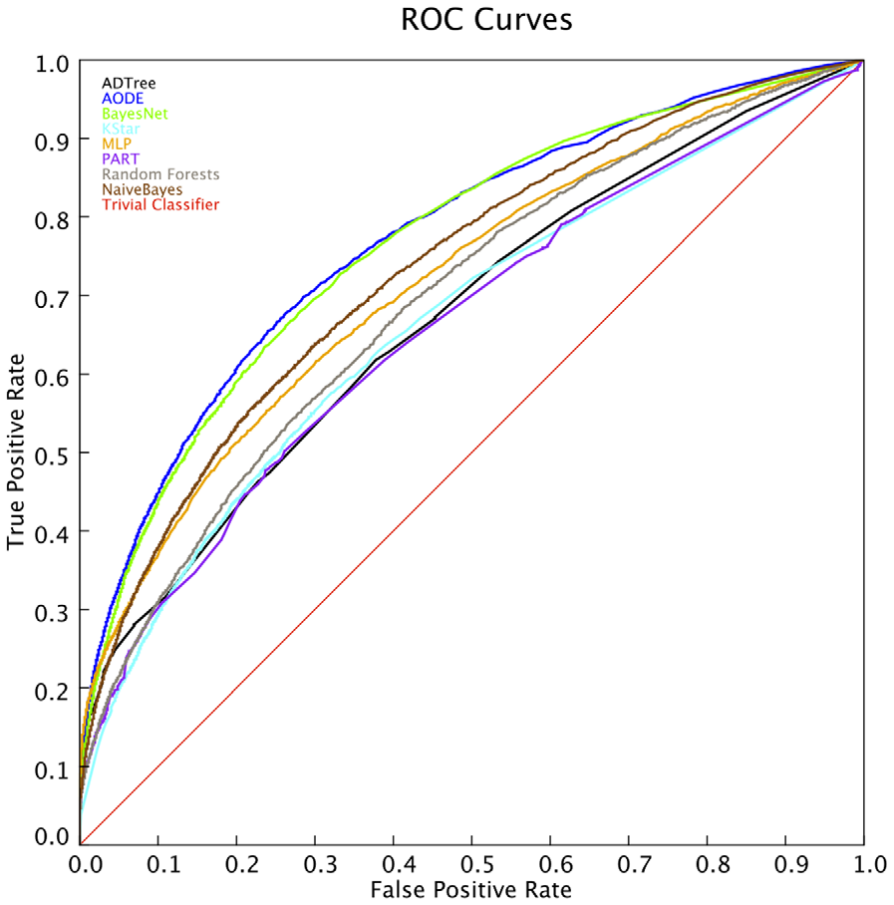
ROC curves for several machine learning algorithms. The X-axis represents the true positive rate and the Y-axis the false positive rate. The legend should be interpreted as in figure 1, with the same order in the algorithms.

For comparative purposes, the results obtained with the widely applied Receiver Operating Characteristic (ROC) are shown (Fig. 2). While ROC analysis leads to the same conclusions and supports the superior performance of Bayesian classifiers to address our problem, no significant differences where found between the two Bayesian approaches adopted that consider features dependence. Briefly summarizing, these two Bayesian classifiers clearly perform better than all the other classifiers tested, although AODE provides a slightly higher performance in some conditions. As a consequence of the results of this test we consider AODE as suitable choice for this problem, even if it is impossible to guaranty that it will be superior to any other classifier in all conditions.

### Improvement by combining different information sources

**Figure 3:**
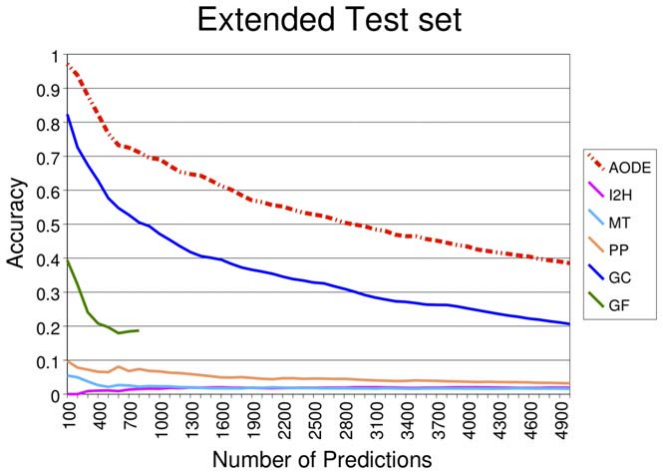
Methods accuracy for the Extended Test Set. The X-axis represents the accumulative number of ‘n’ first predicted interactions, sorted by the corresponding method score, which is different in each case. The Y-axis represents the accuracy, calculated as the ratio of true positives (TP) and total number of predictions considered in the extended test set (all the predictions obtained for *E. coli*, but those used in our training set). *I2H* stands for *in silico* two-hybrid, *MT* for mirrortree, *PP* for phylogenetic profiles, *GC* stands for gene context, *GF* stands for gene fusion and *AODE* for Averaged One Dependence Estimators.

We considered it convenient to compare the accuracy of the positive predictions made by the original methods and the AODE-based classifier (APPIA), since each method has different applicability and is potentially able to detect different type of interactions. We compared the performance of the individual methods with the one of their combination using APPIA by examining the accuracy of the ‘n’ first predictions from an extended test set, that includes the whole set of predictions for *E. coli* after removing those cases used in the training set. In Fig. 3 each line represents the accuracy, measured as the ratio of true positive predictions divided by the number of predictions in the extended test set for an increasing number of predicted pairs (the equivalent figure for the Test Set is shown in Fig. S1). As can be seen, APPIA performs better than each individual method across the entire range of ‘n’ first predictions. As such, APPIA has an accuracy of 0.97 within the first 100 predictions, 0.69 within the first 1000, 0.56 within the first 2000, 0.49 within the first 3000, and so on.

When the comparison was done over a larger set of predicted pairs (we selected the first 800 predictions, that is the total number of predictions provided by the GF method, APPIA was 1.41 times more accurate than the GC method, 3.80 times more accurate than GF, 9.65 times more than PP, 32.38 times more than MT and 47.67 times more accurate than I2H. The results obtained with GC were the closest to APPIA and in fact, both methods define almost the same landscape, although APPIA is 10 or 20 percentage points more accurate. The cause of this difference seems to be the information added by the other individual methods (GF, I2H, MT and PP) and additional attributes such as protein length and size of protein family used in APPIA.

Moreover, we found that some individual methods are very inaccurate (that is less than 10%), such as I2H, MT and PP. By contrast, GC produced a much higher proportion of correct predictions and indeed, GC was about 8 times more accurate than PP (the best of the three poorest methods). GF provided very few predictions due to its dependence on the occurrence of a particular event (gene fusion). The relatively low frequency of gene fusion events limits the ability of GF to predict most interactions.

It is important to notice, that our comprehensive definition of functional associations is focused on the most informative types of associations (see Methods). In our case, the main contributors are co-regulation and metabolic pathways. Therefore, APPIA is expected to be particularly useful for the prediction of these types of associations. Consequently, the advantages of APPIA when compared to other methods are expected to be more important for these cases.

In conclusion, APPIA fusion of the various prediction methods outperformed the individual computational methods by combining and complementing them with additional information such as ranking attributes and biological characteristics (a detailed comparison of features contribution is summarized in Table S1).

### Application to a high throughput dataset

In order to show the potential of APPIA we have applied it to an experimental dataset not included in our training and test sets. For this purpose, we compiled the set of protein complexes detected by Arifuzzaman *et al.* [24]. This data was collected by a high throughput experimental approach based on pull-down technology applied to *E. coli* proteins. It has been shown that high throughput technologies, although valuable, often result in a large number of false positives due to different methodological artifacts. We have chosen this example because it includes a particularly large number of protein associations that cannot be confirmed by any other available data. Indeed, we found that only 7.85% of the data could be confirmed with our comprehensive set of predictions of functional associations (covering 0.64% of these ones). The number of external confirmations is small (a common fact when interaction sources are compared), even though our dataset includes another set of protein complexes from a similar high throughput pull down experiment (Butland et al. [18]).

**Figure 4:**
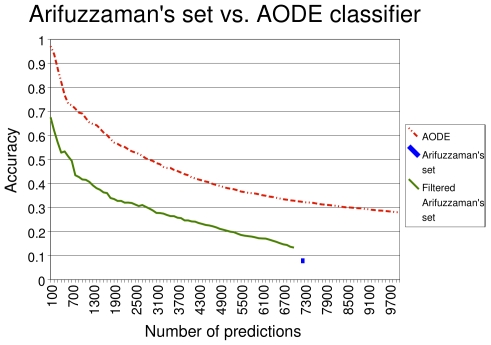
Accuracy of APPIA for the Arifuzzaman experimental set. The X and Y-axis should be interpreted as in figure 3. The filtered set (green line) is obtained sorting the protein pairs in the external database, i.e. the Arifuzzaman set, according the AODE score. Accuracy of the Arifuzzaman set is represented by its mean value (a blue point). This dataset cannot be sorted because there is no associated score.

We used APPIA to detect the subset of potentially biologically meaningful interactions from this large-scale data set. We sorted the set of protein pairs collected by Arifuzzaman using APPIA score as a measure of the likelihood of a functional association, and compared the level of confirmation for the ‘n’ best scoring pairs with the level obtained for the whole Arifuzzaman *data set* and for those pairs predicted for the whole proteome (Fig. 4). The results show clearly that the combination of information provided by APPIA is able to extract a set of significant functional associations from the noisy original data collection. For example, 68% of the first 100 pairs and 42% of the first 1000 ones are confirmed in list ranked with the AODE values. These figures are very significant when compared to the original 8% confirmation for the whole set of 7283 associations in the Arifuzzaman set. When comparing “filtered Arifuzzaman's set” and AODE predictions, it is important to note that the predictions for “filtered Arifuzzaman's set” are less reliable than those for whole proteome, because they include different pairs. In fact, most of the reliable pairs for the whole proteome were not retrieved by the Arifuzzaman's experiment and therefore they couldn't be recovered by APPIA.

These results show the power of combining different data sources and how easily the new predictor can be used to extract valuable functional protein interactions from large-scale experimental protein interaction datasets.

### Comparison with the STRING database

STRING [2] is a database dedicated to the prediction of functional associations between proteins for a set of fully sequenced genomes. It contains an extensive compilation of data ranging from imported external databases to in-house generated predictions, including versions of some of the gene fusion, genome context and phylogenetic profiles methods. STRING has its own definition of a Gold Standard for functional associations based on metabolic pathways [2]. This approach differs from the strategy we adopted because it does not include regulatory relationships between transcription factors and regulated genes, or between the genes regulated by the same transcription factor. Even more importantly, STRING takes advantage of the experimental information available to predict metabolic-like functional associations, providing interesting additional experimental data for a given relationship. By contrast, the predictions produced here are more focused on associations for which there is not available experimentally confirmation, which also implies that in principle our predictions are applicable to any protein (within the *E. coli* proteome here analyzed). Therefore, the coverage and the capacity to discover unknown associations of APPIA should be higher, while its capacity for detecting well-characterized protein associations will be necessarily lower.

**Figure 5:**
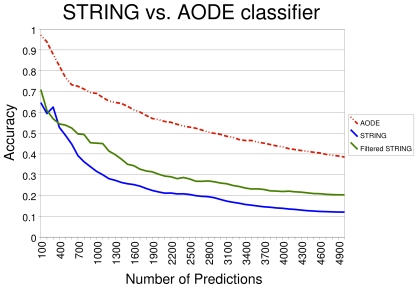
Comparison of APPIA and STRING accuracies on the STRING prediction set. The X and Y-axis should be interpreted as in figures 3 and 4. The filtered set (green line) is obtained sorting the protein pairs in the external database, i.e. STRING, according the AODE score. The STRING line (the blue one) is calculated sorting the data according the STRING score, i.e. the external database score.

In order to test these ideas, we used APPIA to extract those STRING entries with higher scores from a set of 240,885 protein pairs above the minimal STRING confidence value of 0.15. In order to obtain a view of the ability of both approaches for detecting unknown functional associations all the experimentally validated pairs were removed. We compare the STRING and APPIA scores for the set of 121,042 associations present in both datasets, representing 50.25% of the ones in STRING (Fig. 5).

The results obtained clearly show that both definitions of functional links only partially overlap and that APPIA can complement STRING predictions with a more comprehensive definition of functional association between protein pairs.

### EcID server

Results from each of the five prediction methods and from their combination in APPIA are integrated in the EcID server (*E. coli* Interaction Database, [25]), thereby allowing the user to retrieve and navigate easily among the network of functional protein interactions.

EcID supports two basic network navigation modes: the ‘Experimental Mode’ focused on retrieving experimentally supported associations (similar to STRING approach) and the ‘Prediction Mode’ focused on providing predictions for less well-characterized proteins. The APPIA scores presented here are used to generate a prediction confidence criteria for the functional associations displayed in the ‘Prediction Mode’. This allows the system to provide a valuable set of putative protein relationships for otherwise poorly characterized proteins. Moreover, it fulfils our original purpose of gaining further insight into less well-characterized proteins rather than simply ranking well-known protein associations. The experimental server is freely accessible at http://ecid.bioinfo.cnio.es/.

## Discussion

We have presented here a new classifier to predict functional protein associations based on the AODE algorithm. We considered the AODE and BayesNet classification algorithms as the best candidates to address our problem, the prediction of protein functional interactions by combining results of five heterogeneous prediction methods, protein size and number of orthologues of each one of the two proteins. We additionally include as input information the ranking position of the potential interactor in the sorted list of scores of each one of the five methods. These algorithms are particularly suitable for problems containing missing data, as is the case here where it is not always possible to obtain predictions with the five methods for the same protein pairs. AODE and BayesNet have a better capacity to infer the states of unknown variables using prior probabilities and existing evidence. This is an important feature for a classifier thought to be part of a periodically updated pipeline, as is the case of protein/gene databases. Additionally, AODE provides quantitative probability estimates that can be used as a measure of reliability associated to each predicted pair.

This classifier based on the AODE algorithm (APPIA) takes advantage of other computational methods based on the detection of different evolutionary signals. All the methods and information used as the input for the classifier are derived from the analyses of information provided by genomic sequencing experiments. Therefore, APPIA is intended to provide insights into poorly characterized functional associations, rather than highlighting well-known ones. In this sense, our approach differs from other popular and successful approaches, like STRING, because it is independent of the experimental information available for a considered protein pair.

We focused on *E. coli* since it is well characterized at the molecular level, with functions experimentally assigned to approximately 57% of its genes (GenProtEC, February 2007) and with homology-based assignments for an additional 25%. In addition, bacteria are a good workbench due to the quantity of genomes available and the relatively simple architecture of their proteins, both factors that fundamentally boost the quantity and quality of the predictions obtained by genome-based prediction methods. Moreover, at least one of the methods (genomic context) relies on the principle of genome organization that can only be strictly applied to bacterial genomes (conservation of genomic intergene proximity).

We showed that a recently proposed machine-learning algorithm AODE is well suited to detect and rank functional protein associations being particularly valuable for scenarios characterized by a large proportion of missing values. Additionally AODE can be easily and efficiently re-trained, making it a suitable technique for its incorporation into periodically updated resources.

In order to assess the performance of this algorithm, we show the results of the preparatory comparative analysis carried out with alternative machine learning algorithms. To benchmark the predictions obtained by different computational methods we used both “Cost Curves” and ROC analyses. The results of these analyses in a training-independent test set confirmed the suitability of AODE to address this type of problem. Indeed, the AODE-based classifier clearly outperforms all the alternative algorithms evaluated in this test, except the BayesNet classifier that obtains just slightly worse results.

We show that the AODE-based classifier outperforms the original individual methods that it incorporates and that it can be effectively combined with other data sources (experimental or computational) to improve their results. Particularly noteworthy is the combination of our classifier with the results of a previously published large-scale pull down experiment, from which we are able to score the original experimental data according to their functional significance. This result highlights the value of this kind of approach to remove the considerable number of false positives that is still one of the main drawbacks of high-throughput experimental approaches.

In conclusion, our results show that improvement in the prediction of the functional association between proteins can be produced by the integration of computational methods. Furthermore, they emphasize that integrative approaches can be useful to gain insights into proteome characterization by selectively detecting and scoring reliable subsets of functional protein interactions.

## Materials and Methods

There are several difficulties in building a predictor of functional protein interactions due to the implicit nature of the biological data. These difficulties include the intrinsic uncertainty in the data, the extreme imbalance between the number of positive and negative instances (less than 1% of positive class) and the large percentage of values that are missing in relation to several features (99.99% of the instances have at least one attribute without an assigned value, see Table S2).

### Input Data Representation

Each instance (or protein pair) is represented by 19 numerical attributes derived from various sources. We can distinguish three groups of attributes: i) the scores from each of the 5 methods to predict protein interactions (see below); ii) the protein-centred predictions rank for the protein pair, calculated as the position of the protein pair in the sorted list of predictions from each method for each of the two proteins (see below); and iii) 2 pairs of protein features that are highly related to the performance of these methods, the sequence length and the number of orthologues detected for each protein in the pair (see below). In all cases, the values missing for any of the 19 attributes were considered as ‘undetermined’ values and they were not replaced with flags because in our situation the absence of information cannot be considered as usable information. Although using flags provides similar results (see Table S1) we prefer the alternative implementation that has a slightly better positive recovering rate and handles missing data directly.

#### Computational prediction methods

The computational prediction methods used are based on different sources of evidence suggesting an interaction between a pair of proteins (Valencia and Pazos, 2002). The inputs for the classifier presented here includes the scores for each of the following 5 prediction methods:

1. Phylogenetic Profiles (PP), a method to examine the presence or absence of genes in related species [3]. The score used was calculated according to the original publication. It is based on the Hamming distance of the vectors presence or absence of an ortholog for every *E. coli* protein in each of the compiled genomes.
2. Gene Context (GC) considers the conservation of the gene neighborhoods in different species [4]. The score was calculated as the number of cases in which the orthologues of the gene pair of *E. coli* were at a genomic distance of closer than 300 bp in the corresponding chromosome, according to the original publication.
3. Gene Fusion (GF) searches for non-overlapping similarity matches within the same protein of pairs of proteins [5]–[6]. In this case we use the z-scores collected from http://cgg.ebi.ac.uk/services/allfuse/.
4. Mirror Tree (MT) studies the similarity of phylogenetic trees [11] and the MT score involves the Pearson's correlation coefficient of the all versus all orthologues sequence distance matrices for the pair of proteins studied.
5. *In silico* Two-Hybrid (I2H) quantifies the degree of co-variation between pairs of residues in the two proteins [10], and it was implemented in accordance with the original publication.

All the methods (except GF that came from an external source) were applied to a set of 118 complete prokaryotic genomes (see Table S3). For the in-house methods, only reciprocal best BLAST [26] hits between *E. coli* and the corresponding genome were selected as putative orthologues (both e-values were required to be smaller than 1E-5).

I2H and MT require pairs of multiple sequence alignments for their application. We generated the corresponding multiple sequence alignment for each set of orthologues with more than 15 sequences using MUSCLE [27]. Thus, we built 2183 alignments, each containing the orthologous sequences detected for a different *E. coli* protein.

It is important to note that all the methods are used in their original formats. We preferred to keep these simpler formats and to enrich the input with the related attributes. This way, the training algorithm itself can be optimized for these characteristics.

#### Protein-centered ranks of predictions

Several of the methods are expected to have some protein-related biases (e.g. some phylogenetic profiles are more usual and, therefore, PP over predicts associations among the corresponding proteins), while each input pair is considered to be independent of any other in our algorithms. To cope with this situation, we calculated the ranking position of the corresponding protein pair in the sorted prediction lists for each of the two proteins in every method. Thus, for each protein we introduced the values of the smallest and the highest ranking of the pair in the five prediction methods (i.e. 10 additional attributes). These methods provide a large number of low scoring results. Therefore we also removed the entries where none of the 10 rankings were less than or equal to 100, only for PP, MT and I2H rankings. This step reduces the noise coming from uninformative pairs that would never be predicted by the input methods

#### Protein features

Finally, we included 4 attributes that represent two very basic protein features that are intrinsically related with the performance of one or more of the prediction methods. These features are the number of orthologues detected and the sequence length for each protein in the pair.

### Building the various datasets

A particularly controversial issue when making predictions of protein interaction is the definition of a functional association. In our case, we have chosen an inclusive definition of functional association that is consistent with the different prediction methods included. While some of these methods are expected to focus on physical interactions (GF, I2H or MT), others are better suited to predict biochemical pathways (GC) or they have a less well-defined scope (PP). Indeed, our definitions of positive and negative classes are an expansion of the datasets used in our recently proposed new protein interaction method [25]. These datasets have been completed with functional information not necessarily related to physical protein interactions, such as regulatory information (see below).

#### Positive class

The set of functional associations for E. coli proteins was extracted from several external databases (see below) and this set tries to capture the complex nature of the domain. It contains 89,401 different protein pairs (homodimers were not considered) from the following sources:

1. Proteins involved in the same biochemical pathway. These functional associations are extracted from the KEGG14 [28] and EcoCyc15 [29] databases. We considered all the proteins assigned to the same pathway to be functionally associated by pairs, even though this does not necessarily imply a direct physical interaction between them. We obtained 20,860 associations from KEGG and 3,446 from EcoCyc.
2. Regulator-regulated gene associations. We extracted the transcription regulatory data contained in EcoCyc and established functional links between each transcriptional regulator and its corresponding regulated genes. This set contains 1,686 relationships.
3. Set of co-regulated genes. Based on the same type of information as the previous set, we established 58,275 functional associations among those proteins that are regulated by the same regulator.
4. Interactions directly extracted from the literature using text-mining techniques. For this set we use the set of protein name interactions obtained from iHOP for *E. coli* proteins [30]. These interactions are defined as the mention of proteins in the same sentence of PubMed abstracts. In this way, we retrieved 6,686 text mining-based interactions.
5. Set of physical interactions derived from low-throughput experiments. We used collections of 401, 58 and 2684 physical interactions for *E. coli* proteins annotated in: DIP ([31], http://dip.doe-mbi.ucla.edu/); BIND ([32], http://www.bind.ca/Action); and IntAct ([33], http://www.ebi.ac.uk/intact/), respectively.
6. Collection of protein complexes extracted from EcoCyc. These complexes are based on manual curation of the scientific literature and they represent a high quality set of very well known complexes. We established a functional link for each pair of proteins that are part of the same complex. This resulted in a set containing 950 protein associations.
7. Protein complexes extracted from Butland et al. [18]. This high throughput pull-down experiment provides information similar to the set of complexes from EcoCyc (although less reliable). This set includes 4,745 associations and it is expected to have greater sensitivity and lower specificity than the previous one.

This definition of positives is intended to be a comprehensive representation of the functional associations among proteins. As a consequence of the different amount of information available for each type of functional associations, both training and test analyses are going to be more informative about the predictive capabilities of different methods for the main contributors to this set of positives, i.e. co-regulated genes and metabolic associations.

#### Negative class

We generated the negative set from the non-positive pairs among the proteins contained in the positive set (homodimers were not considered). Thus, we tried to reduce the uncertainty in the negative information by considering only proteins with information available regarding protein function. From the remaining pairs, we removed those pairs for which the prediction methods generated no value, because they are uninformative for our classifier. This process yielded 2,575,779 negative protein pairs. It is important to note that this set may still contain some uncharacterized functional associations.

#### Datasets

Building training and test sets must deal with the problem of the imbalance between classes (the negative class initially constitutes over 99% of all the instances). Thus, the training and test datasets were built using 20% positive and 80% negative instances as a compromise between representing the underlying distribution and providing a more balanced detail of both classes without affecting classification performance.

The training set was composed of 2/3rds of the positive instances (making up the 20% explained above), while the test set included the remaining 1/3 of the positive protein pairs. The training and test sets were completed with some of the instances from the negative class and these were exactly 4 times the number of positive instances, to reach 80% of negative instances in each set as indicated previously. Hence, all the positive instances available were employed in the training or test set. By contrast, many negative instances were discarded. Hence, according to the aforementioned criteria, the test set was half the size of the corresponding training set.

### Learning Algorithms

We use AODE algorithm for the classification [17]. AODE achieves classification by averaging over all of a small space of alternative Naïve-Bayes-like models that have weaker independence assumptions than NB [22]. This modification is intended to avoid bias with a very small increase in variance. Resulting algorithm relaxes the attribute independence assumption increasing prediction accuracy and maintaining computational efficiency.

AODE is inspired by the notion of n-dependence estimators [34]. An ‘n’-dependence estimator is similar to NB except that each attribute depends upon at most ‘n’ other attributes, in addition to the class. NB is a zero-dependence estimator, unlike the well-known TAN, which is a one-dependence estimator [20].

Higher-dependence estimators typically have a weaker bias but a higher variance than NB. AODE avoids training time computation and reduces variance by overall averaging of a limited class of one-dependence estimators, since the more effective ones also typically have a very high computational complexity for training time [17] (for a more detailed description of AODE, see Text S1). AODE demands nominal attributes and therefore, discretization was performed using the “equal frequency binning” criterion, with a minimum of 50 instances per band. This criterion was chosen because it presents the best empirical results against other possibilities, such as “equal width binning” (data not shown).

AODE handles missing values by using only the known values of each instance when it calculates the product of probabilities. This idea is suitable for our domain, because it neither fills in unknown attribute values with the mean (or median value), nor does it ignore the instance completely like some other algorithms. Filling missing values with the mean or majority value (as BayesNet [20]–[21] and Naïve Bayes do [22]) does not reflect the semantics in our data, as features with a missing value might imply they do not exist. Likewise, ignoring missing attribute values would not be viable in our domain either, as almost all the instances have some missing value (only 82 complete instances among 2,665,180). This is due to the fact that the computational methods only give a result in constrained conditions, so as to reach a minimum number of orthologues in the proteins or to trigger an event (see methods descriptions [35]).

For comparison, the preparatory phase test performed using several machine learning methods has been included. The algorithms included are: decision trees (in its new version ADTree [36]); case based reasoning (Kstar [37]); neural networks (MultiLayer Perceptron, MLP [38]–[39]); rules decision (PART [40]); random forests [23], whose efficiency is proved in other similar domains [15]; and another Bayesian method (BayesNet [20]–[21]).

Besides AODE, BayesNet is a relevant algorithm in this study. BayesNet refers to Bayesian Network [20]. The architecture and implementation used here are the weka's default ones [21]. This algorithm implies the learning of the network structure and the learning of the probability tables. A hill climbing learning algorithm called K2 [41] is used to infer the network structure. This algorithm adds arcs with a fixed ordering of variables. Assessment of the quality of the learned network is done using a Bayesian metric [21]. Direct estimates of the conditional probability distribution tables of the Bayes network are done with a simple estimator [21]. There is a modification in the default configuration, referred to the maximum number of parents a node can have in the net structure, fixing it to 2. Thus, a Tree Augmented Bayes Network (TAN) is learned.

We used Weka's implementation [42] for all the machine learning algorithms applied here.

### Assessment Method

In order to assess the performance of the different classifiers we used Cost Curves (Curve Tool [19]) that were generated from the results obtained with each classifier for the test set described below (see Figure 1).

A Cost Curve analysis is a graphical technique used to visualize the performance (error rate or expected cost) of binary classifiers over the full range of possible class distributions and misclassification costs. In a simpler interpretation, Cost Curve plots represent the cost probability, equivalent [19] to the percentage of positive instances in the data set to which the classifier is applied, versus the normalized expected cost, equivalent to the ratio of the mistakes both in terms of false positives as well as false negatives. This interpretation assumes that the cost of misclassifying positive examples (i.e. FN) is the same as the cost of misclassifying negative examples (i.e. FP) [19]. When the cost of misclassifying is different, the X-axis does not only represent the fraction of positive instances, but the product of the cost of misclassifying and the probability of an instance being from the positive class. Y-axis indicates the fraction of the difference between the maximum and the minimum possible costs that will be incurred when the classifier is used [19]. Thus, Y-axis shows the normalized expected cost for the cost scenario and class distribution show by the value in the X-axis. On the left hand side of the plot the curves measure the increasing ratio of false positives (FP), while on the right hand side it shows the decreasing ratio of true positives (TP).

Accordingly, the corresponding cost curve of a classifier is made up of different straight lines, with the extremes at both sides of the Y-axis and corresponding to several pairs <ratio FP, ratio TP> obtained for the different classification thresholds. The line segments that are not dominated by any other (i.e., the lowest ones) make up the whole cost curve.

This representation usually contains the curve corresponding to the trivial classifier (red line in Figure 1), which always assigns the same class to any instance as if it were a random classification. In these graphs the best classifier is that with the lower curve (lower cost) and the Cost Curves for useful classifiers should always be below the trivial classifier curve, in order to be a good classifier for whatever distribution between positive and negative instance class in the dataset. Accordingly, the points in a curve that intersects the trivial classifier (if they exist) determine the range of the X-axis for which it is not suitable to use such a classifier since simple random chance performs better. As a generic rule, a valid curve has probability cost values lower than 0.3 (in Y-axis).

The major advantage of Cost Curves over ROCs is that they allow a direct read out of the performance for any specific combination of misclassification and class distribution. At the same time, they show directly how performance changes across the full range of values.

The correspondence between these two graphical techniques is that one point of the former corresponds to one line in the latter. The co-ordinates of a point in the ROC are the left hand and right hand extremes in the Y-axis of the cost curve. Each line in a cost curve consists of many classifiers that come from two variables: different thresholds and different numbers of positive instances in the data set. As in the ROC curve, the threshold of Cost Curves determines the cut-off between the positive and negative class. However, Cost Curves show more detailed information about performance than the ROC curve with respect to class distribution, because it only has one point and not a line to represent the performance according to the different positive and negative class distributions.

Another positive characteristic of the cost curves is that they allow various classifiers to be readily compared. These classifiers could be generated by applying assorted machine learning algorithms or from different training and/or test sets. Thus, in Cost Curves the error difference between a pair of classifiers can be automatically measured through the vertical distance, which is not so easy in ROC curve [19].

In summary, a Cost Curve is equivalent to a ROC in their information content and they can be inter-converted. In this case Cost Curves are used because they are easier to interpret in meaningful units and they facilitate the selection of the best classifier by simple visualization under certain conditions, for example, for the cost of misclassification and probability of a specific class.

## Supporting Information

Figure S1 Methods accuracy for the Test Set. The X-axis represents the accumulative number of ‘n’ first predicted interactions, sorted by the corresponding method score, which is different in each case. The Y-axis represents the accuracy, calculated as the ratio of true positives (TP) and total number of predictions considered in the test set extracted from our gold standard of functional associations (see Methods). I2H stands for in silico two-hybrid, MT for mirrortree, PP for phylogenetic profiles, GC stands for gene context, GF stands for gene fusion and AODE for Averaged One Dependence Estimators.
(0.15 MB TIF)

Table S1 Performance of different classifiers for the Test Set. This table shows performance related descriptors for a number of different classifiers. The descriptors included are: Area Under the ROC Curve (AUC), Mathews Correlation Coefficient (MCC, formula shown below), True Positives (TP), True Negatives (TN), False Positives (FP) and False Negatives (FN). The table is divided in three regimes. The first one (yellow background) represents the incremental inclusion of features in AODE classifiers. New features are included from the most to the least discriminative (MCC score for these features) ones: Methods (Gene Fusion, Gene Context, Phylogenetic Profiles, Mirror Tree and in silico two-hybrid), Length (protein sequence lengths) and Nseqs (number of sequences). Rankings in the list of scores for each method are finally included (as they are derived from the corresponding methods) to build the presented APPIA classifier. The second regime (white background) shows the performance for the AODE using all the features and with flags instead of missing values. Finally, the third regime (green background) shows the performance of the other seven different classifying algorithms used in the preliminary test. MCC = (TP×TN−FP×FN)/SQRT((TP + FN)×(TP + FP)×(TN + FP)×(TN + FN)).
(0.02 MB PDF)

Table S2 Attributes statistics. This table shows some statistical measures of each attribute used in the classification process. The range of the column values represents the minimum and maximum value reached for this attribute in all the examples. In each case, the mean and the standard deviation are calculated without taking into account the instances with an unknown value. Total number of instances: 2,665,180. It should be noted that the high percentage of unknown values is important in many attributes.
(0.06 MB PDF)

Table S3 List of fully sequenced genomes used. This is the set of 118 prokaryotic genomes to which the computational prediction method has been applied.
(0.04 MB PDF)

Text S1 AODE detailed description. A detailed description of the AODE algorithm.
(0.07 MB PDF)

## References

[1] Mering Cv, Krause R, Snel B, Cornell M, Oliver SG (2002). Comparative assessment of large-scale data sets of protein-protein interactions.. Nature.

[2] Mering Cv, Huynen M, Jaeggi D, Schmidt S, Bork P (2003). STRING: A database of predicted functional associations between proteins.. Nucleic Acids Res.

[3] Pellegrini M, Marcotte E, Thompson M, Eisenberg D, Yeates T (1999). Assigning protein functions by comparative genome analysis: Protein phylogenetic profiles.. PNAS.

[4] Dandekar T, Snel B, Huynen M, Bork P (1998). Conservation of gene order: A fingerprint of proteins that physically interact.. Trends in Biochemical Sciences.

[5] Enright A, Iliopoulos I, Kyrpides N, Ouzounis CA (1999). Protein interaction maps for complete genomes based on gene fusion events.. Nature.

[6] Marcotte EM, Pellegrini M, Ng HL, Rice DW, Yeates TO (1999). Detecting protein function and protein-protein interactions from genome sequences.. Science.

[7] Bowers P, Cokus S, Eisenberg D, Yeates T (2004). Use of logic relationships to decipher protein network organization.. Science.

[8] Morett E, Korbel J, Rajan E, SaabRincon G, Olvera L (2003). Systematic discovery of analogous enzymes in thiamin biosynthesis.. Nat Biotechnol.

[9] Wu J, Kasif S, DeLisi C (2003). Identification of functional links between genes using phylogenetic profiles.. Bioinformatics.

[10] Pazos F, Valencia A (2002). In silico two-hybrid system for the selection of physically interacting protein pairs.. Proteins.

[11] Pazos F, Valencia A (2001). Similarity of phylogenetic trees as indicator of protein-protein interaction.. Protein Eng.

[12] Juan D, Pazos F, Valencia A (2008). High-confidence prediction of global interactomes based on genome-wide coevolutionary networks.. PNAS.

[13] Pazos F, Ranea JAG, Juan D, Sternberg MJE (2005). Assessing protein co-evolution in the context of the tree of life assists in the prediction of the interactome.. J Mol Biol.

[14] Sato T, Yamanishi Y, Kanehisa M, Toh H (2005). The inference of protein-protein interactions by co-evolutionary analysis is improved by excluding the information about the phylogenetic relationships.. Bioinformatics.

[15] Qi Y, Bar-Joseph Z, Klein-Seetharaman J (2006). Evaluation of different biological data and computational classification methods for use in protein interaction prediction.. Proteins: Structure, Function, and Bioinformatics.

[16] Lu LJ, Xia Y, Paccanaro A, Yu H, Gerstein M (2005). Assessing the limits of genomic data integration for predicting protein networks.. Genome Res.

[17] Webb GI, Boughton JR, Wang Z (2005). Not so naive bayes: Aggregating one-dependence estimators.. Mach Learn.

[18] Butland G, Peregrin-Alvarez JM, Li J, Yang W, Yang X (2005). Interaction network containing conserved and essential protein complexes in escherichia coli.. Nature.

[19] Drummond C, Holte RC (2006). Cost curves: An improved method for visualizing classifier performance.. Mach Learn.

[20] Friedman N, Geiger D, Goldszmidt M (1997). Bayesian network classifiers.. Mach Learning.

[21] Bouckaert RR (2004). http://weka.sourceforge.net/manuals/weka.bn.pdf.

[22] John GH, Langley P (1995). Estimating continuous distributions in bayesian classifiers..

[23] Breiman L (2001). Random forests.. Mach Learn.

[24] Arifuzzaman M, Maeda M, Itoh A, Nishikata K, Takita C (2006). Large-scale identification of protein-protein interaction of escherichia coli K-12.. Genome Res.

[25] León EA, Ezkurdia I, García B, Valencia A, Juan D (2009). EcID. A database for the inference of functional interactions in E. coli.. Nucl Acids Res.

[26] Altschul SF, Gish W, Miller W, Myers EW, Lipman DJ (1990). Basic local alignment search tool.. J Mol Biol.

[27] Edgar RC (2004). MUSCLE: Multiple sequence alignment with high accuracy and high throughput.. Nucleic Acids Res.

[28] Kanehisa M, Goto S, Hattori M, Aoki-Kinoshita KF, Itoh M (2006). From genomics to chemical genomics: New developments in KEGG.. Nucleic Acids Res.

[29] Keseler IM, Collado-Vides J, Gama-Castro S, Ingraham J, Paley S (2005). EcoCyc: A comprehensive database resource for escherichia coli.. Nucleic Acids Res.

[30] Hoffmann R, Valencia A (2004). A gene network for navigating the literature.. Nat Genet.

[31] Salwinski L, Miller CS, Smith AJ, Pettit FK, Bowie JU (2004). The database of interacting proteins: 2004 update.. Nucleic Acids Res.

[32] Alfarano C, Andrade CE, Anthony K, Bahroos N, Bajec M (2005). The biomolecular interaction network database and related tools 2005 update.. Nucl Acids Res.

[33] Hermjakob H, Montecchi-Palazzi L, Lewington C, Mudali S, Kerrien S (2004). IntAct: An open source molecular interaction database.. Nucleic Acids Res.

[34] Sahami M (1996). Learning limited dependence bayesian classifiers..

[35] Valencia A, Pazos F (2002). Computational methods for the prediction of protein interactions.. Curr Opin Struct Biol.

[36] Freund Y, Mason L (1999). The alternating decision tree learning algorithm..

[37] Cleary JG, Trigg LE (1995). K*: An instance-based learner using an entropic distance measure..

[38] Bishop CM (1995). Neural networks for pattern recognition..

[39] Rumelhart DE, McClelland JL (1986). Parallel distributed processing..

[40] Frank E, Witten IH (1998). Generating accurate rule sets without global optimization..

[41] Cooper GF, Herskovits E (1992). A bayesian method for the induction of probabilistic networks from data.. Mach Learn.

[42] Witten IH, Frank E (2005). Data mining: Practical machine learning tools and techniques..

