# Supplementary figures and images for: Inference of Functional Relations in Predicted Protein Networks with a Machine Learning Approach
Source: PLoS One. 2010 Apr 1;5(4):e9969. doi: 10.1371/journal.pone.0009969 (PMC2848617; doi:10.1371/journal.pone.0009969)

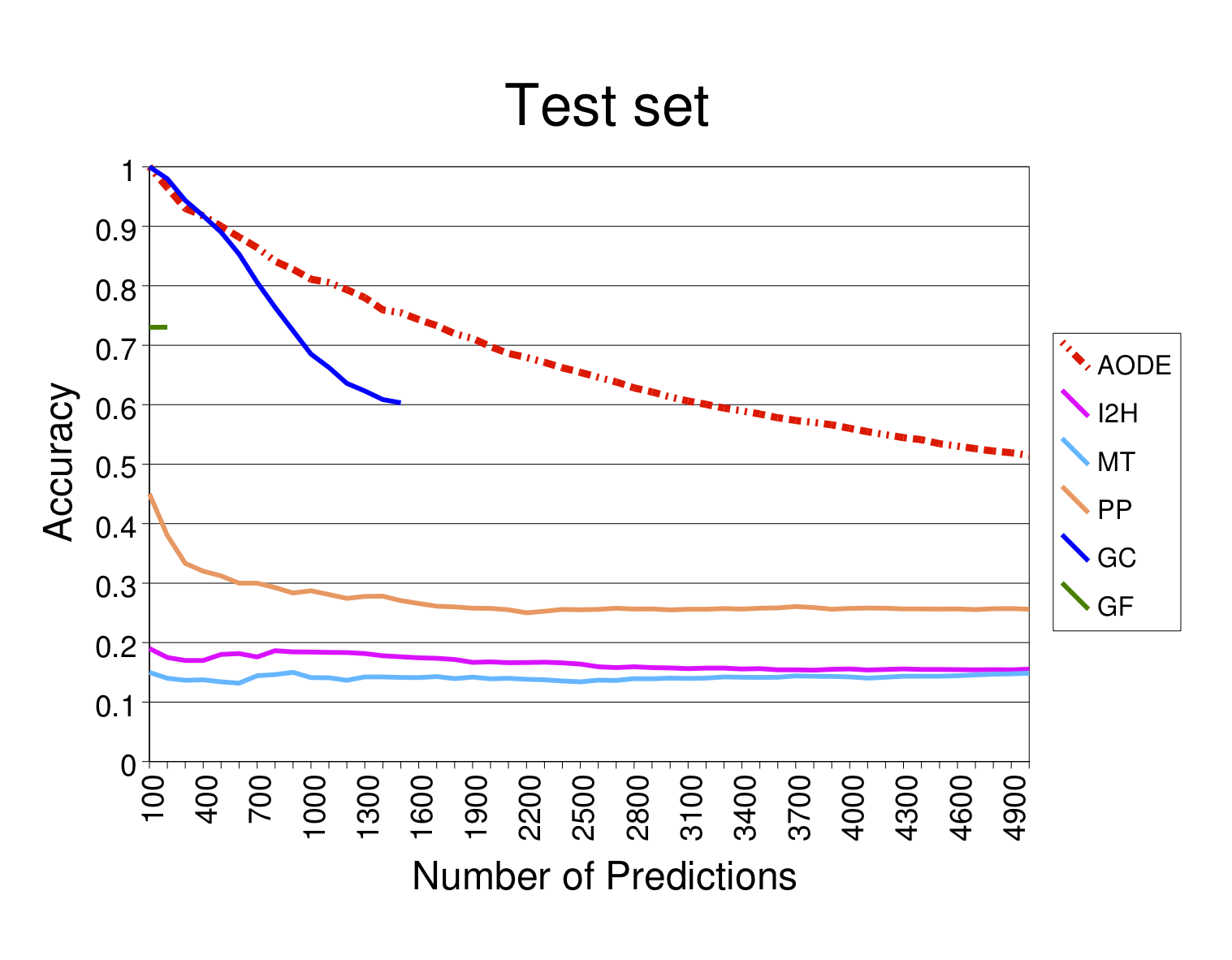

Supplement: Figure S1 — Methods accuracy for the Test Set. The X-axis represents the accumulative number of ‘n’ first predicted interactions, sorted by the corresponding method score, which is different in each case. The Y-axis represents the accuracy, calculated as the ratio of true positives (TP) and total number of predictions considered in the test set extracted from our gold standard of functional associations (see Methods). I2H stands for in silico two-hybrid, MT for mirrortree, PP for phylogenetic profiles, GC stands for gene context, GF stands for gene fusion and AODE for Averaged One Dependence Estimators. (0.15 MB TIF) [file pone.0009969.s001.tif]
