# Supplementary material for: Inference of Functional Relations in Predicted Protein Networks with a Machine Learning Approach
Source: PLoS One. 2010 Apr 1;5(4):e9969. doi: 10.1371/journal.pone.0009969 (PMC2848617; doi:10.1371/journal.pone.0009969)
Supplement: Table S1 — Performance of different classifiers for the Test Set. This table shows performance related descriptors for a number of different classifiers. The descriptors included are: Area Under the ROC Curve (AUC), Mathews Correlation Coefficient (MCC, formula shown below), True Positives (TP), True Negatives (TN), False Positives (FP) and False Negatives (FN). The table is divided in three regimes. The first one (yellow background) represents the incremental inclusion of features in AODE classifiers. New features are included from the most to the least discriminative (MCC score for these features) ones: Methods (Gene Fusion, Gene Context, Phylogenetic Profiles, Mirror Tree and in silico two-hybrid), Length (protein sequence lengths) and Nseqs (number of sequences). Rankings in the list of scores for each method are finally included (as they are derived from the corresponding methods) to build the presented APPIA classifier. The second regime (white background) shows the performance for the AODE using all the features and with flags instead of missing values. Finally, the third regime (green background) shows the performance of the other seven different classifying algorithms used in the preliminary test. MCC = (TP×TN−FP×FN)/SQRT((TP + FN)×(TP + FP)×(TN + FP)×(TN + FN)). (0.02 MB PDF) [file pone.0009969.s002.pdf]

Table S1: Alternative Classifiers

Features	AUC	MCC	TP	TN	FP	FN
all (APPIA)	0.77	0.35	1362	21523	4160	565
Methods, Length, Nseqs	0.77	0.27	903	21685	4619	403
Methods, Length	0.67	0.18	542	21714	4980	374
Methods	0.57	0.14	305	21903	5217	185
Length	0.67	0.13	306	21843	5216	245
nseqs	0.61	0.06	146	21891	5376	197
APPIA (with Flags)	0.79	0.34	1206	21671	4316	417
**Algorithm**	**AUC**	**MCC**	**TP**	**TN**	**FP**	**FN**
BayesNet	0.77	0.34	1674	21020	3848	1068
Naive Bayes	0.73	0.13	1419	19097	4103	2991
MLP	0.72	0.33	1050	21784	4472	304
Random Forests	0.69	0.24	1159	21006	4363	1082
KStar	0.67	0.22	1962	18979	3560	3109
ADTree	0.67	0.30	910	21793	4612	295
PART	0.66	0.22	481	21948	5041	140
