# Supplementary material for: Inference of Functional Relations in Predicted Protein Networks with a Machine Learning Approach
Source: PLoS One. 2010 Apr 1;5(4):e9969. doi: 10.1371/journal.pone.0009969 (PMC2848617; doi:10.1371/journal.pone.0009969)
Supplement: Table S2 — Attributes statistics. This table shows some statistical measures of each attribute used in the classification process. The range of the column values represents the minimum and maximum value reached for this attribute in all the examples. In each case, the mean and the standard deviation are calculated without taking into account the instances with an unknown value. Total number of instances: 2,665,180. It should be noted that the high percentage of unknown values is important in many attributes. (0.06 MB PDF) [file pone.0009969.s003.pdf]

Table S2: Attributes Statistics

(Total instances: 2665180. The mean and the standard deviation are calculated without taking into account instances with unknown values.)

Attribute	Known / Unknown Values	Values Range	Mean	Standard Deviation
**I2H**	1054149 / 1611031 (39.55% / 60.45%)	[0, 35.349]	0.771	0.585
**MT**	1054149 / 1611031 (39.55% / 60.45%)	[0, 0.991]	0.615	0.194
**PP**	2591226 / 73954 (97.23% / 2.77%)	[0.088, 1]	0.646	0.168
**GC**	11690 / 2653490 (0.44% / 99.56%)	[1, 145]	3.651	8.093
**GF**	668 / 2664512 (0.03% / 99.97%)	[1, 157]	7.266	18.837
**n_seqs_min**	1594687 / 1070493 (59.83% / 40.17%)	[16, 113]	332.729	16.646
**n_seqs_max**	2523554 / 141626 (94.69% / 5.31%)	[16, 113]	55.957	26.614
**length_seq_min**	2665180 / 0 (100.00% / 0.00%)	[24, 1538]	248.032	118.405
**length_seq_max**	2665180 / 0 (100.00% / 0.00%)	[46, 2003]	459.751	215.676
**pos_rank_I2H_min**	1054149 / 1611031 (39.55% / 60.45%)	[1, 2168]	615.329	465.049
**pos_rank_I2H_max**	1054149 / 1611031 (39.55% / 60.45%)	[1, 2181]	998.099	545.625
**pos_rank_MT_min**	1054149 / 1611031 (39.55% / 60.45%)	[1, 2137]	543.095	396.529
**pos_rank_MT_max**	1054149 / 1611031 (39.55% / 60.45%)	[1, 2181]	1039.962	535.107
**pos_rank_PP_min**	2591226 / 73954 (97.23% / 2.77%)	[1, 2946]	1125.718	769.455
**pos_rank_PP_max**	2591226 / 73954 (97.23% / 2.77%)	[1, 2946]	1877.786	798.469
**pos_rank_GC_min**	11690 / 2653490 (0.44% / 99.56%)	[1, 20]	4.275	2.895
**pos_rank_GC_max**	11690 / 2653490 (0.44% / 99.56%)	[1, 23]	7.588	4.563
**pos_rank_GF_min**	668 / 2664512 (0.03% / 99.97%)	[1, 11]	1.121	0.549
**pos_rank_GF_max**	668 / 2664512 (0.03% / 99.97%)	[1, 25]	2.867	3.104
