# Supplementary material for: Inference of Functional Relations in Predicted Protein Networks with a Machine Learning Approach
Source: PLoS One. 2010 Apr 1;5(4):e9969. doi: 10.1371/journal.pone.0009969 (PMC2848617; doi:10.1371/journal.pone.0009969)
Supplement: Table S3 — List of fully sequenced genomes used. This is the set of 118 prokaryotic genomes to which the computational prediction method has been applied. (0.04 MB PDF) [file pone.0009969.s004.pdf]

List of fully sequenced genomes used

Acinetobacter sp.
Aeropyrum pernix
Anabaena sp.
Anaplasma marginale
Aquifex aeolicus
Archaeoglobus fulgidus
Azoarcus sp.
Bacillus cereus ATCC 10987
Bacillus licheniformis Goettingen
Bacillus licheniformis Novozymes
Bacteroides thetaiotaomicron
Bartonella henselae
Bdellovibrio bacteriovorus
Bifidobacterium longum
Bordetella bronchiseptica
Borrelia burgdorferi
Bradyrhizobium japonicum
Brucella suis
Buchnera aphidicola Acyrthosiphon pisum
Burkholderia mallei
Burkholderia pseudomallei
Campylobacter jejuni NCTC 11168
Candidatus Blochmannia floridanus
Caulobacter crescentus
Chlamydia pneumoniae TW-183
Chlamydia trachomatis
Chlorobium tepidum
Chromobacterium violaceum
Clostridium acetobutylicum
Corynebacterium glutamicum Kalinowski
Corynebacterium glutamicum Nakagawa
Coxiella burnetii
Deinococcus radiodurans
Desulfotalea psychrophila
Desulfovibrio vulgaris
Enterococcus faecalis
Erwinia carotovora
Escherichia coli K12
Francisella tularensis
Fusobacterium nucleatum
Geobacillus kaustophilus
Geobacter sulfurreducens
Gloeobacter violaceus
Gluconobacter oxydans
Haemophilus influenzae ATCC 51907
Haloarcula marismortui
Halobacterium salinarium
Helicobacter hepaticus
Idiomarina loihiensis
Lactobacillus plantarum
Lactococcus lactis

Legionella pneumophila Philadelphia 1
Leifsonia xyli
Leptospira interrogans lai
Listeria innocua
Mannheimia succiniciproducens
Mesoplasma florum
Methanobacterium thermoautotrophicum
Methanococcus jannaschii
Methanococcus maripaludis
Methanopyrus kandleri
Methanosarcina acetivorans
Methylococcus capsulatus
Mycobacterium leprae
Mycobacterium paratuberculosis
Mycobacterium tuberculosis Oshkosh
Mycoplasma penetrans
Nanoarchaeum equitans
Neisseria meningitidis A
Nitrosomonas europaea
Nocardia farcinica
Oceanobacillus iheyensis
Onion yellows phytoplasma
Parachlamydia sp.
Pasteurella multocida
Photobacterium profundum
Photorhabdus luminescens
Picrophilus torridus
Porphyromonas gingivalis
Prochlorococcus marinus MIT 9313
Propionibacterium acnes
Pseudomonas aeruginosa
Pyrobaculum aerophilum
Pyrococcus horikoshii
Ralstonia solanacearum
Rhizobium loti
Rhizobium meliloti
Rhodopirellula baltica
Rhodopseudomonas palustris
Rickettsia conorii
Rickettsia typhi
Salmonella typhi CT18
Shewanella oneidensis
Shigella flexneri 301
Silicibacter pomeroyi
Staphylococcus aureus Mu50
Staphylococcus epidermidis ATCC 35984
Streptococcus agalactiae V
Streptomyces coelicolor
Sulfolobus solfataricus
Symbiobacterium thermophilum
Synechococcus sp. WH8102
Synechocystis sp.

Thermoanaerobacter tengcongensis
Thermoplasma volcanium
Thermotoga maritima
Thermus thermophilus HB27
Treponema denticola
Tropheryma whipplei Twist
Ureaplasma parvum
Vibrio vulnificus YJ016
Wigglesworthia glossinidia brevipalpis
Wolbachia pipientis wMel
Wolinella succinogenes
Xanthomonas axonopodis
Xylella fastidiosa 9a5c
Yersinia pseudotuberculosis
Zymomonas mobilis
