# Supplementary material for: Inference of Functional Relations in Predicted Protein Networks with a Machine Learning Approach
Source: PLoS One. 2010 Apr 1;5(4):e9969. doi: 10.1371/journal.pone.0009969 (PMC2848617; doi:10.1371/journal.pone.0009969)
Supplement: Text S1 — AODE detailed description. A detailed description of the AODE algorithm. (0.07 MB PDF) [file pone.0009969.s005.pdf]

Text S1: AODE detailed description

AODE (Averaged One-Dependence Estimators) [1] is a machine learning classifier.

This algorithm improves Naïve Bayes (NB) model [2] relaxing the attribute independence assumption of NB, where each attribute can depend on the class.
The Naïve Bayes algorithm is based on conditional probabilities defined in Eq. 1, where $y \in c_1, ..., c_k$ are the k classes of an example $x = <x_1, . . . , x_n>$, where x_i is the value of the ith attribute.

$$P(y|x) = P(y,x)/P(x) \qquad \text{(Eq. 1)}$$

Hence, the value class for an example is calculated according Eq. 2, it means the highest conditional probabilities accumulation of each attribute respect to class attribute.

$$\underset{y}{\operatorname{argmax}}\left(\hat{P}(y)\prod_{i=1}^{n}\hat{P}(x_i|y)\right) \qquad \text{(Eq. 2)}$$

where $\hat{P}(y)$ and $P(x_i|y)$ are estimates of the respective probabilities derived from the frequency of their respective arguments in the training sample. At training time NB just needs to compile a table of class probability estimates and a table of conditional attribute-value probability estimates, from the training set [1].

AODE improves independence assumptions, without increasing computational complexity. Naïve Bayes approaches, such as Lazy Bayes Rules [3] are very costly in classification time, while other approaches like TAN [4] require higher training times. AODE achieves lower computational cost by replacing model selection with model average. Classes are calculated by aggregating predictions from an ensemble of one-dependence classifiers by aggregating the predictions of all qualified classifiers, as Eq. 3 shows.

$$\underset{y}{\operatorname{argmax}}\left(\sum_{i:1\le i\le n \wedge F(x_i)\ge m}\hat{P}(y,x_i)\prod_{j=1}^{n}\hat{P}(x_j|y,x_i)\right) \qquad \text{(Eq. 3)}$$

If $\neg\exists i : 1\le i \le n \wedge F(x_i) \ge m$, AODE defaults to NB. $F(x_i)$ is a count of the number of training examples having x_i value-attribute.

In this experiment we used the weka's AODE implementation [5].

References

1. Webb, G. I., Boughton, J. R. & Wang, Z. Not So Naive Bayes: Aggregating One-Dependence Estimators. Mach. Learn. 58, 5-24 (2005).

2. John, G. H. &Langley, P. Estimating Continuous Distributions in Bayesian Classifiers (Proceedings of the Eleventh Conference on Uncertainty in Artificial Intelligence, Morgan Kaufmann, San Mateo, 1995).

3. Zheng, Z., & Webb, G. I. (2000). Lazy learning of Bayesian Rules. Machine Learning, 41 (1), 53–84.

4. Friedman, N., Geiger, D. & Goldszmidt, M. Bayesian Network Classifiers. Mach. Learning 29, 131-163 (1997).Witten, I. H. & Frank, E. in Data Mining: Practical machine learning tools and techniques (ed Kaufmann, M.) (Morgan Kaufmann, San Francisco, 2005).
